# 

**Published:** 1842-10

**Authors:** 


### CONTENTS OF No. XXVIII.

OF THE

### British and Foreign Medical Review.

OCTOBER, 1842.

### Part First.—Analytical and Critical Reviews.

PAGE

Art. I.—1. The Spas of Germany. By A. B. Granville, m.d. . . 309
2. Pilgrimages to the Spas. By James Johnson, m.d. . . ib.
3. The Mineral Springs of England. By Edwin Lee, Esq. . . ib.
4. A Practical Treatise on the Efficacy of Mineral Waters in the Cure of Chronic Diseases. By Sir A. M. Downie, m.d. . . . . ib.
5. The Sanative Influence of Climate. By Sir J. Clark, Bart. . . ib.
6. Rapport fait à l'Académie Royale de Médecine sur les Eau Minérales de France pendant les années 1834-5-6. Par M. F. V. Merat, rapporteur . ib.
7. Theoretisch-praktisches Handbuch der Heilquellenlehre. Von Aug. Vetter ib.
8. Marienbad, et ses différents moyens curatifs dans les Maladies Chroniques. By C. J. Heidler, m.d. . . . . . . ib.
9. Ueber den Gebrauch der Mineralquellen, im besondere derer zu Ems. Von Dr. J. Vogler . . . . . . . ib.

Art. II.—Ueber Darm-Anhangs-Brüche (Herniæ Litricæ). Mit Bemerkungen über Kothfisteln und Widernatürlichen After. Von Dr. C. F. Riecke 360

Art. III.—1. Traité Pratique de l'Art des Accouchemens. Par Chailly, d.m. 366

2. On the Theory and Practice of Midwifery. By Fleetwood Churchill, m.d. ib.

Art. IV.—Samuel Thomas von Soemmering vom Baue des menschlichen Körpers. Zweiter Band: Knochen-und Bänderlehre. Dritter Band: Muskel-und Gefasslehre. Vierter Band: Anatomie des Gehirns und Nervensystems 373

Art. V.—1. De la Menstruation; faire connaitre l'influence que cette fonction exerce sur les Maladies, et celle qu'elle en reçoit. Par A. Brierre de Boismont, d.m. 382
2. De la Menstruation considérée dans ses rapports Physiologiques et Pathologiques. Par A. Brierre de Boismont. . . . . . ib.

Art. VI.—Trans. of the Provincial Medical and Surgical Association. Vol. X. 396

Art. VII.—Ueber das Verhältniss der Medicin zur Chirurgie; und die Duplicität im ärtzlichen Stande, &c. Von Dr. Ph. Fr. von Walther . . 402

Art. VIII.—Die rheumatischen Krankheiten, nach ihrem Wesen, &c. Von Georg Friedr. Greiner, m.d. . . . . . . 412

Art. IX.—1. Hydropathy, or the Cold Water Cure, as practised by Vincent Preissnitz at Graeffenberg, Silesia, Austria. By R. T. Claridge, Esq. . . 422
2. A Practical Treatise on the Cure of Diseases by Water, Air, Exercise, and Diet, &c. By James Wilson . . . . . ib.

Art. X.—The Pharmacopœia of the United States of America . . 437

Art. XI.—1. Der Leberthran als Heilmittel, auf Grundlage vielfacher Thatsachen und Versuche an Thieren. Von Dr. Hermann Klencke . 441
2. Beobachtungen über den Leberthran, besonders in seiner Anwendung gegen scrophulöse Krankheitsformen. Von Eduard Adolph Panck . ib.

Art. XII.—1. Hygiène philosophique; ou de la Santé dans le Régime physique, moral, et politique de la Civilisation moderne. Par J. J. Virey, m.d. 446
2. Essai d'Hygiène générale. Par L. C. A. Motard . . . ib.
3. The Principles of Population, and their Connexion with Human Happiness. By Archibald Alison, f.r.s. . . . . . ib.

Art. XIII.—Recherches Cliniques sur le Liquide Céphalo-Rachidien ou Cerebro-Spinal. Par F. Magendie . . . . . . . 462

Art. XIV.—The Climate of the South of Devon, and its Influence upon Health. By Thomas Shapter, m.d. . . . 470

§

PAGE

Art. XV.—1. Allgemeine Anatomie. Lehre von den Mischungs-und Formbestandtheilen des menschlichen Körpers. Von J. Henle . . . 478
2. Handbuch der allgemeinen Anatomie des Menschen und der Haussäugethiere. Von Fr. Gerber . . . . . . ib.
3. Elements of the General and Minute Anatomy of Man and the Mammalia. By Fr. Gerber . . . . . . ib.
4. Lehrbuch der allgemeinen Anatomie des Menschen. Von Victor Bruns . ib.
5. Anatomie Microscopique. Par le Docteur L. Mandl . . ib.
6. Entwurf eines neuen genetischen Systems der Histologie, zugleich als Grundriss einer philosophischen Anatomie. Von Dr. Hermann Klencke . ib.
7. On the Employment of the Microscope in Medical Studies. By Dr. J. H. Bennett ib.

Art. XVI.—Animal Chemistry; or Organic Chemistry in its applications to Physiology and Pathology. By Justus Liebig, m.d. p.h.d. f.r.s. m.r.i.a. 492

Art. XVII.—Observations on Life, as the cause of the Vital Phenomena . 521

### Part Second.—Bibliographical Notices.

Art. I.—The Nervous System and its Functions. By Herbert Mayo, f.r.s. . 525

Art. II.—The Education of Mothers of Families. By M. Aime-Martin . 527

Art. III.—An Introductory Lecture on Pictorial Anatomy. By J. Miller, f.r.s.e. 528

Art. IV.—Die Lehre von der Zurechnungsfähigkeit bei Zweifelhaften Gemüthszuständen für Aerzte und Juristen praktisch dargestellt. Von Dr. A. Schnitzer 529

Art. V.—De Methodi Endermaticæ Ratione nec non applicatione. Auctore Ferdinando Schubert . . . . . 530

Art. VI.—Analekten für Frauenkrankheiten, etc. . . . 531

Art. VII.—Memoir of the late James Hope, m.d. By Mrs. Hope . . 532

Art. VIII.—A Treatise on Irritation of the Spinal Nerves. By J. E. Riadore, m.d. 533

Art. IX.—Traité des Maladies des Femmes qui déterminent des Fleurs Blanches, des Leucorrhées, ou tout autre Ecoulement Utéro-Vaginal. Par Henry Blatin et V. Nivet . . . . . . 534

Art. X.—G. Vrolik ueber eine Vollkommene Verwachsung der Gelenke an den Kreuz-Darm-und Schaambeinen ohne vorhergegangene krankhafte Beschaffenheit . . . . . . . 535

Art. XI.—The Biographical Dictionary of the Society for the Diffusion of Useful Knowledge. Part I. A. . . . . . . 536

Art. XII.—A Bedside Manual of Physical Diagnosis. By Charles Cowan, m.d. ib.

Art. XIII.—Pulmonary Consumption. By Henry Gilbert, m.r.c.s. . ib.

Art. XIV.—A Collection of Plates illustrating the Theory and Practice of Midwifery; accompanied by an Explanatory Text. By Em. Debout, m.d. 538

Art. XV.—An Essay on Diabetes. By H. Bell, d.m.p. . . . ib.

Art. XVI.—Conradi Joannis Martini Langenbeck, Icones Anatomicæ . 539

Art. XVII.—The Anatomy of the Urinary Bladder and Perinæum of the Male. By Alexander Monro, m.d. f.r.s.e., &c. . . . 540

### Part Third.—Selections from the British and Foreign Journals.

### I. THE FOREIGN JOURNALS.

#### ANATOMY AND PHYSIOLOGY.

M. Bourgery on the minute Structure of the Spleen . . . 541
Dr. Joseph Engel on Fatty and Cellular Degeneration of Muscle . . 543
on the Pathology of the Tissues . . . 544
Observations confirming the Experiments of Panizza on the Nerves of the Tongue 545
M. Bourgery on the minute Structure of the Lungs in Man and the Mammalia . 546
M. Noel Gueneau de Mussy on the Muscular Coat of the Stomach . . ib.
M. Mandl on the Development of the Entozoa . . . 547

### PATHOLOGY, PRACTICAL MEDICINE, AND THERAPEUTICS.

PAGE
Dr. Helmbrecht on a Case of Laceration of the Pulmonary Artery . 547
Dr. Budge on Softening of the Brain . . . . . . ib.
Dr. Winternitz on Monomania Ophthalmica . . . 548
Dr. Bredschneider on Rupture of the Trachea . . . . 549
Prof. G. Barilli on Tic Douloureux from a Tumour beneath the Cerebellum . ib.
Dr. Beonhardi on Peritonitis . . . . . ib.
Dr. Hartmann on apparent Death by Lightning . . . . 550
Dr. Negrier on a very simple means of arresting Epistaxis . . ib.
Prof. Seidlitz on Typhus and Typhus Abdominalis . . . 551
M. Serres on the Insect Origin of Smallpox . . . 553
Dr. Bicking on a Case of suddenly-occurring and as suddenly-ceasing Dumbness ib.
M. Piorry on a peculiar Granular Appearance of the Buffy Coat of the Blood . ib.

### SURGERY.

M. J. Parise on Spontaneous or Symptomatic Luxations of the Femur . . 554
M. Bertherand on a new Cranial Saw . . . . 555
Dr. Asmus on the Passage of Air into the Veins . . . ib.
M. Berard on Intra-parietal Hernia after a Wound of the Abdomen . . 556
Dr. Frantz on Strangulated Hernia through the foramen thyroideum . ib.
Prof. Retzius on a remarkable Case of Thoracic Fistula . . ib.
On the Surgical use of the Magnet . . . . 557
M. Dumeril on the Preservation of Nitrate of Silver . . . ib.
M. Blandin on Exophthalmia, with Œdema of the Conjunctiva, and Opacity of the Crystalline Lens in a Puerperal Woman . . . ib.
M. A. Berard on Fibrous Polypi of the Uterus . . . . 558
Dr. Holscher on the Treatment of Leucoma by Incisions into the Cornea . ib.
M. Lauer on the local Employment of Calomel in Ophthalmia Neonatorum . 559

### MIDWIFERY, AND DISEASES OF WOMEN AND CHILDREN.

Prof. Osiander on the Management of Cases of Prolapsus of the Funis . ib.
M. Trousseau on the Employment of Quinine in the Diseases of Children . 560
M. J. Aubry on extra-uterine Pregnancy in the Patient's 70th year . . ib.
Dr. Russegger on a singular Case . . . . 561

### MEDICAL STATISTICS.

Statistics of Delirium Tremens in Berlin . . . . . ib.

---

### II. THE AMERICAN AND COLONIAL JOURNALS.

### ANATOMY AND PHYSIOLOGY.

Dr. W. Muller on Evolution of Electricity . . . . . 562

### PATHOLOGY, PRACTICAL MEDICINE, AND THERAPEUTICS.

J. Pagan, Esq. on Chronic Pleuritis, with Ossification of the Diaphragm . ib.

### SURGERY.

Dr. Ridley on Cancer of the Penis . . . . . 563
Dr. Burns on Iodine in Erysipelas . . . . . . ib.

### MIDWIFERY, AND DISEASES OF WOMEN AND CHILDREN.

Dr. Marsh on Atresia Vaginæ . . . . . . ib.

---

### III. THE BRITISH JOURNALS, &c.

### ANATOMY AND PHYSIOLOGY.

Messrs. Goodsir and Bowman on the Physiology of Secretion and Absorption . 564
Mr. Shaw on the best Mode of conducting Physiological Researches . 568

PAGE
Mr. Gulliver on the smallest Blood-corpuscles of Mammals . . 570
Figure of the Oval Blood-corpuscles of Vertebrata . 571
Lymph-globules of Birds . . . . ib.
Muscular Fibres of the Gullet . . . ib.
Nuclei of the Blood-corpuscles of Vertebrata . 572
Structure of Fibrin . . . . ib.
Character of the Spermatozoa, Molecules of the Semen ib.
Dr. Davis on Millipedes discharged from the Stomach . . . ib.
Dr. Rees on Analysis of the Contents of the Thoracic Duct in the Human Subject 573
Mr. Harrison on Extrophy of the Bladder . . . . ib.
Mr. Addison on Distribution of Air-passages, and Formation of Air-cells in the Lungs ib.
Mr. Paget on the relative Sizes of the Trunks and Branches of Arteries . ib.
Mr. Robertson on the Period of Puberty in Negro Women . . ib.
Mr. Renaud on Corpora Lutea . . . . . . ib.
Mr. Erichsen on the Influence of coronary Circulation on the Action of the Heart 575
Dr. Helland on the Forces by which the Blood is circulated in the capillary Vessels ib.

### PATHOLOGY, PRACTICAL MEDICINE, AND THERAPEUTICS.

Mr. Markwick on Rhubarb as an external Application in sloughing Venereal Ulcers 576
Dr. Bird on Pathological Chemistry . . . . . . ib.
Mr. Allen on an Ulcer of the Thorax, communicating with the right Lung . ib.
Mr. Johnson on Death from Peas . . . . . . ib.
Dr. Kennion on Perforation of the Stomach . . . . . 577
Mr. Miller on the Treatment of Hemorrhagic Diathesis . . . ib.
Mr. Lowdell on an interesting Case of Syphilis . . . 578.
Mr. Robinson on Albuminuria . . . . . . ib.
Dr. Allan on Hemorrhagic Diathesis . . . . . ib.
Mr. Barker on an Abscess in the Lungs, pointing below the Umbilicus . 579
Mr. Allan on Fæcal Vomiting during thirty-four days . . ib.
Mr. Gillat on the post-mortem Appearances of a Case of Grinders' Asthma . 580

### SURGERY.

Dr. Byron on Malignant Diseases of the Head and Face . . ib.
Mr. Williamson on Displacement of Stomach and Colon in consequence of a Wound ib.
Mr. Hargreaves on Osseous Aneurism . . . . . 581
Mr. Coulson on Mr. Stafford's Treatment of Stricture of the Urethra . ib.
Mr. Lyell on Morphia in Strangulated Hernia . . . . ib.
Mr. Leney on Moxa in Rheumatism . . . . . ib.
Mr. Farr on Tincture of Catechu in Sore Nipple . . . . 582

### MIDWIFERY, AND DISEASES OF WOMEN AND CHILDREN.

Dr. Jamieson on an extraordinary Case of Twins . . . ib.
Dr. Thomson on a Case of Short Funis . . . . ib.
Dr. Lynn on a Case of Arm Presentation, in which Turning was impossible . ib.
Mr. Collyns on a Case of Uterine Polypus and Short Funis . . 583
Dr. David on a Case of Triplets . . . . . . ib.
Mr. Bredon on a large Congenital Tumour . . . . ib.

### MEDICAL JURISPRUDENCE AND TOXICOLOGY.

Dr. Spittal on a Case of Suicide . . . . . . ib.
Dr. Scoffern on Poisoning by Sulphuric Acid . . . . 584
Dr. Renaud on Corpora Lutea . . . . . . ib.

---

### Part Fourth.—Medical Intelligence.

Mr. Wharton Jones on the Anatomy, Physiology, and Pathology of the Blood . 585
Books received for review . . . . . . 601
Title, Index, &c.

